# The effects of interleukin-8 on airway smooth muscle contraction in cystic fibrosis

**DOI:** 10.1186/1465-9921-9-76

**Published:** 2008-12-01

**Authors:** Vasanthi Govindaraju, Marie-Claire Michoud, Pasquale Ferraro, Janine Arkinson, Katherine Safka, Hector Valderrama-Carvajal, James G Martin

**Affiliations:** 1 Seymour Heisler Laboratory of the Montreal Chest Institute Research Center and Meakins Christie Laboratories, McGill University, Montreal, Quebec, Canada; 2 University of Montreal Hospital Center, Montreal, Quebec, Canada

## Abstract

**Background:**

Many cystic fibrosis (CF) patients display airway hyperresponsiveness and have symptoms of asthma such as cough, wheezing and reversible airway obstruction. Chronic airway bacterial colonization, associated with neutrophilic inflammation and high levels of interleukin-8 (IL-8) is also a common occurrence in these patients. The aim of this work was to determine the responsiveness of airway smooth muscle to IL-8 in CF patients compared to non-CF individuals.

**Methods:**

Experiments were conducted on cultured ASM cells harvested from subjects with and without CF (control subjects). Cells from the 2^nd ^to 5^th ^passage were studied. Expression of the IL-8 receptors CXCR1 and CXCR2 was assessed by flow cytometry. The cell response to IL-8 was determined by measuring intracellular calcium concentration ([Ca^2+^]_i_), cell contraction, migration and proliferation.

**Results:**

The IL-8 receptors CXCR1 and CXCR2 were expressed in both non-CF and CF ASM cells to a comparable extent. IL-8 (100 nM) induced a peak Ca^2+ ^release that was higher in control than in CF cells: 228 ± 7 versus 198 ± 10 nM (p < 0.05). IL-8 induced contraction was greater in CF cells compared to control. Furthermore, IL-8 exposure resulted in greater phosphorylation of myosin light chain (MLC_20_) in CF than in control cells. In addition, MLC_20 _expression was also increased in CF cells. Exposure to IL-8 induced migration and proliferation of both groups of ASM cells but was not different between CF and non-CF cells.

**Conclusion:**

ASM cells of CF patients are more contractile to IL-8 than non-CF ASM cells. This enhanced contractility may be due to an increase in the amount of contractile protein MLC_20_. Higher expression of MLC_20 _by CF cells could contribute to airway hyperresponsiveness to IL-8 in CF patients.

## Background

Cystic fibrosis (CF) is a genetic disease caused by defective Cl^- ^secretion and enhanced Na^+ ^absorption across the airway epithelium [1]. The airways become infected with *P. aeruginosa *[2], *S. aureus*, *H*. *influenzae*, and respiratory syncytial virus [3-5]. Chronic bacterial infections and inflammation of the lung are the main causes of morbidity and mortality in CF patients [6]. With increasing age, CF patients develop airway obstruction and many of these patients also suffer from airway hyperresponsiveness and asthma-like symptoms [7,8]. Furthermore, Tiddens et al [9] have shown that airway remodeling similar to that of asthma affects CF airways, including changes in airway smooth muscle. In addition, i*n vivo *studies with inhalation of bronchodilators improve the symptoms associated with bronchial responsiveness in CF patients indicating the presence of an asthma-like syndrome [10-12]. These findings suggest that the bronchial responsiveness observed in CF may be related to an increase in airway smooth muscle (ASM) contraction.

Many inflammatory cytokines are produced in the airways in CF patients [13]. Several studies have documented increased levels of interleukin-8 (IL-8; CXCL8) in bronchoalveolar lavage fluid and sputum and increased expression of IL-8 by bronchial glands of patients with CF [14-16]. In CF affected lungs, IL-8 is produced by neutrophils, airway epithelial cells, macrophages, and monocytes [17]. IL-8 binds to the G-protein coupled receptors CXCR1 and CXCR2 [18]. It acts as a chemotactic agent for neutrophils, T lymphocytes [19], basophils [20], NK cells and melanocytes [21]. It has also been shown that IL-8 stimulates the proliferation and migration of rat vascular smooth muscle [22,23]. IL-8 inhalation provokes bronchoconstriction in guinea pigs *in vivo *[24]. As IL-8 is increased in the airways of CF patients and its action is not restricted to immune effector cells, it is possible that IL-8 may be involved in the airway hyperresponsiveness of CF by increasing smooth muscle contraction. Consistent with this hypothesis, we have demonstrated that ASM from healthy individuals expresses CXCR1 and CXCR2 and that IL-8 increases intracellular [Ca^2+^] and triggers contraction [25]. Therefore, we hypothesized that, given the prolonged exposure of CF ASM to IL-8 *in vivo*, IL-8 may evoke different contractile responses of ASM cells in CF. Thus we investigated the effects of IL-8 on the release of intracellular Ca^2+ ^by ASM and on the contraction of ASM from CF-affected subjects and compared our findings to those of cells from CF non-affected subjects. We also examined the expression of CXCRs and the effects of IL-8 on cellular migration and on ASM cell proliferation in both control and CF-affected subjects.

## Materials and methods

### Cell cultures

Fragments of lobar bronchi were obtained from donors and recipients from lung transplants. The tissue was cut into small pieces of about 5 mm x 5 mm and digested for 90 min at 37°C in Hanks balanced salt solution (HBSS) containing in mM: KCl 5, KH_2_PO_4 _0.3, NaCl 138, NaHCO_3 _4, Na_2_HPO_4 _5.6 to which collagenase type IV (0.4 mg/ml), soybean trypsin inhibitor (1 mg/ml) and elastase type IV (0.38 mg/ml) had been added. The dissociated cells were collected by filtration through 125 μm Nytex mesh and the resulting suspension collected by centrifugation. The pellet was then reconstituted in growth medium (DMEM-Ham's F12 medium supplemented with 10% fetal bovine serum, penicillin 10000 unit/ml, streptomycin 10 mg/ml, and amphotericin 25 μg/ml) and plated in 25-cm^2 ^flasks. ASM cells from CF subjects were isolated and cultured using a modification of the technique described by Randell et al [26] to avoid contamination. Briefly, small pieces of tissue were incubated for 20 minutes in cold Hanks buffer containing 0.5 mg/ml dithiothreitol and 10 μl/ml of Dnase type I, then placed in a cell dissociation medium HBSS containing: 0.4 mg/ml collagenase type IV, 1 mg/ml soybean trypsin inhibitor and 0.38 mg/ml elastase (type IV), penicillin (100 U/ml), streptomycin (100 μg/ml), ceftazidime (100 μl/ml), ciprofloxacin (20 μl/ml), colistin (5 μg/ml), tobramycin (80 μg/ml) and gentamycin: (50 μg/ml. The tissue was digested for 90 minutes at 37°C and the resulting cell suspension filtered and plated as described above. The same antibiotics were added to the culture medium for 48–72 hours. ASM cells in primary cultures were identified by immunostaining for smooth muscle cell specific α-actin, and Western blotting for myosin light chain kinase and calponin.

Confluent cells were detached with 0.025% trypsin solution containing 0.02% ethylenediaminetetraacetic acid (EDTA) and grown on 25 mm diameter glass coverslips for single cell imaging of Ca^2+ ^transients, contraction studies and on 6 well culture dishes for flow cytometry, protein extraction, and chemotaxis assays.

### Contraction studies

ASM cells from CF and non CF individuals were grown for 4 days, in parallel, on glass slides covered with homologous cell substrate as previously described [27]. The glass slides were placed in a Leiden chamber where the temperature was maintained at 37 ± 0.5°C using a temperature controller (model TC-102; Medical System Corp). The cells were visualized using an inverted microscope with 20× magnification using Nomarski optics. A CCD camera (Hamamatsu C2400) was used to acquire and record images (Photon Technology International Inc, Princeton, NJ). Images were taken before and 10 minutes after the addition of IL-8 or phosphate buffered saline (PBS) as a vehicle for IL-8. Images were digitized and analyzed with the Scion software (National Institutes of Health, Bethesda, MD). The length of the cell was measured along its long axis by an observer blinded to the treatment. Contraction was expressed as the percentage decrease in cell length from the initial value.

### Flow Cytometry

ASM cells were incubated with fluorescent labeled antibodies to CXCR1 and CXR2. The cells were fixed and analyzed by flow cytometry (FACScalibur) with commercial software to determine the levels of surface expression of CXCR1 and CXCR2.

### Measurement of intracellular Ca^2+^

Cytosolic Ca^2+ ^was measured using Fura-2 and dual wavelength microfluorimetry. in single cells by imaging a group of 10–15 cells with a CCD camera (Photon Technology Inc, Princeton, NJ) at a single emission wavelength (510 nm) with double excitatory wavelengths (345 and 380 nm) as previously described [28].

### Protein extraction and immunoblotting

Expression and phosphorylation of the regulatory myosin light chain (MLC_20_) were quantified by immunoblotting. Proteins were extracted from IL-8 or vehicle stimulated cells. Blots were developed by chemiluminescence and the signals were acquired with an image analyser. Signals were analyzed by densitometry using commercial software and Imager (Fluorochem™, Flowgen Bioscience Limited, Nottingham, U.K).

### Chemotaxis assay

Chemotaxis assays were performed using a modified Boyden chamber (Neuroprobe, Cabin John, MD). The number of migrated cells following treatments was expressed as a multiple of the value obtained with vehicle treated cells studied on the same day.

### Cell proliferation assay

ASM cells from CF and control subjects were seeded onto six well plates at a density of 3 × 10^4 ^cells per well in DMEM/10% FBS. When the cultures reached 70% confluence, the cells were growth arrested for 48 hours with 0.5% FBS. The agonists, IL-8 (100 nM) and PDGF (10 ng/ml), were then added to the cultures. Forty-eight hours later, the cells were detached and counted on a haemacytometer.

### Data analysis

Data are represented as mean ± SEM unless otherwise indicated. Comparison of means was performed with Student-t tests. One-way ANOVA followed by Student's t-test was used for the chemotaxis assay. The empirical frequency distributions of the contractions of cells in response to IL-8 were compared using a Kolmogorov-Smirnoff test. A difference was considered to be statistically significant when the *P *value was less than 0.05.

## Results

### Effects of IL-8 on contraction of ASM from CF individuals

**Figure 1:**
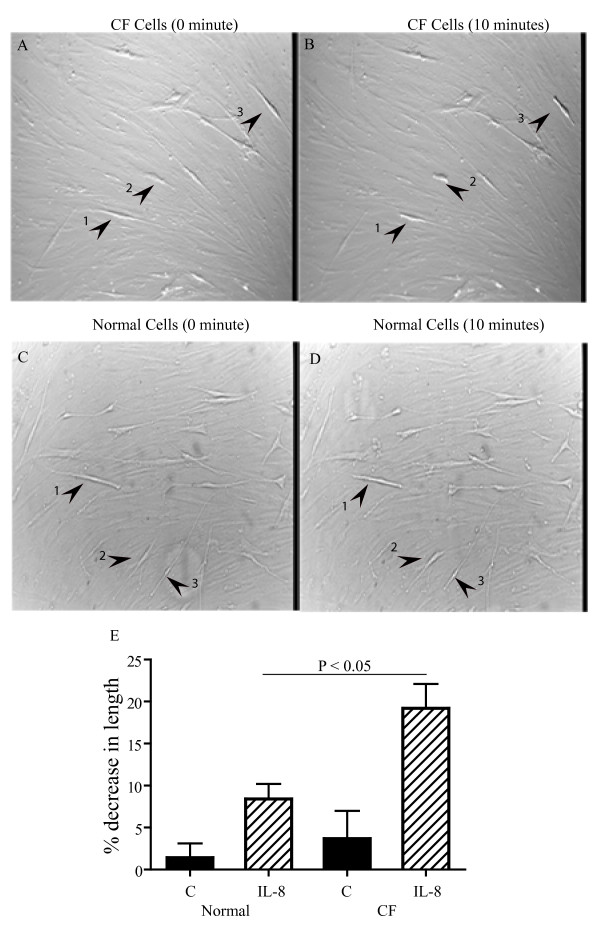
**Contraction of CF and control ASM cells treated with IL-8**. Panels A and B show the images recorded before and 10 minutes after the addition of IL-8 (100 nM) in CF cells. The cells that are clearly visualized are live cells and the indistinct cells are the background of alcohol-fixed cells that serve as a substratum. Arrows indicate the contracted cells. Panel C and D show the images of control cells before and after the addition of IL-8. Panel E represents the % decrease in the CF and non-CF cell lengths (C) following IL-8 or PBS treatments. Cumulative frequency distributions are shown and the distributions were compared statistically using the Kolmogorov-Smirnoff test. The IL-8 treated CF cells shortened to a significantly greater degree than the non-CF cells (P < 0.05). 40 CF cells and 36 control cells from four different individuals per group were measured. The values are expressed in % decrease in the length of the cell following IL-8 stimulation.

The length of the cells was measured before (Figure 1, panels A and C) and at 10 minutes after the addition of IL-8 (Figure 1, panels B and D) to CF and control cells respectively. Resting length was not significantly different between the two groups: CF: 2.84 ± 0.25 vs control: 2.26 ± 0.29 arbitrary units (p = 0.137). The effects of IL-8 and PBS on the lengths of CF and non-CF cells are illustrated as cumulative frequency distributions (Figure 1E). IL-8 (100 nM) significantly decreased the length of the CF cells by 19 ± 3% compared to 8 ± 2% in control cells (p <0.05) whereas the changes in length of control and CF cells treated with vehicle (1.5 ± 1% and 3.7 ± 3%, respectively) did not differ significantly.

### Flow cytometric quantification of CXCR1 and CXCR2

**Figure 2:**
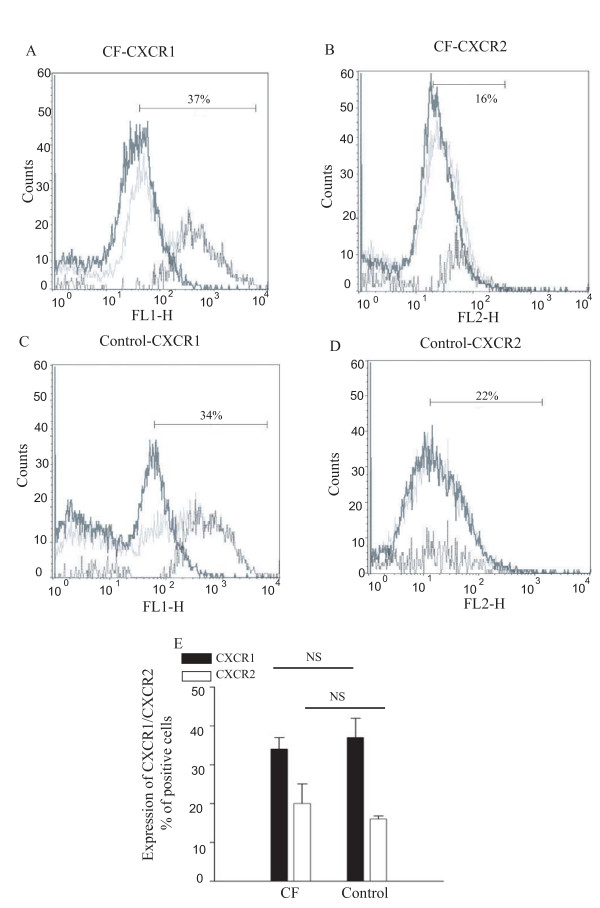
**Flow cytometric analysis of the surface expression of CXCR-1 and CXCR-2 on ASM cells from CF and control patients**. Representative examples of the expression of CXCR1 and CXCR2 from CF and control patients are shown in panels 2A, 2B, 2C and 2D respectively. The histogram outlined by the darkest lines represents the distribution of isotype control cells, the lightest shade represents the cells stained with specific antibody and the intermediate shade represents the difference between positively stained cells and isotype controls. Panel E shows the percentage of cells stained for CXCR1 and CXCR2 from 4 different cell preparations of CF and control patients.

The surface expression of CXCR1 and CXCR2 protein on ASM cells from both control and CF subjects was studied by flow cytometry. The results are presented as overlaid histograms and the percentages of positive cells were calculated by subtraction of isotype controls from antibody marked cells. Figure 2 shows illustrative results of flow cytometry for CF (panel A) and control cells (panel B) for CXCR1, and CF (panel C) and control cells (panel D) for CXCR2. Panel E shows summary data expressed as the % of cells stained for CXCR1 and CXCR2 in CF (37 ± 2% and 16 ± 0.8%, respectively) and control groups (34 ± 2% and 22 ± 2%, respectively). There are no significant differences in the expression of either CXCR1 or CXCR2 by control and CF ASM cells.

### Effects of IL-8 on [Ca^2+^]_i_

**Figure 3:**
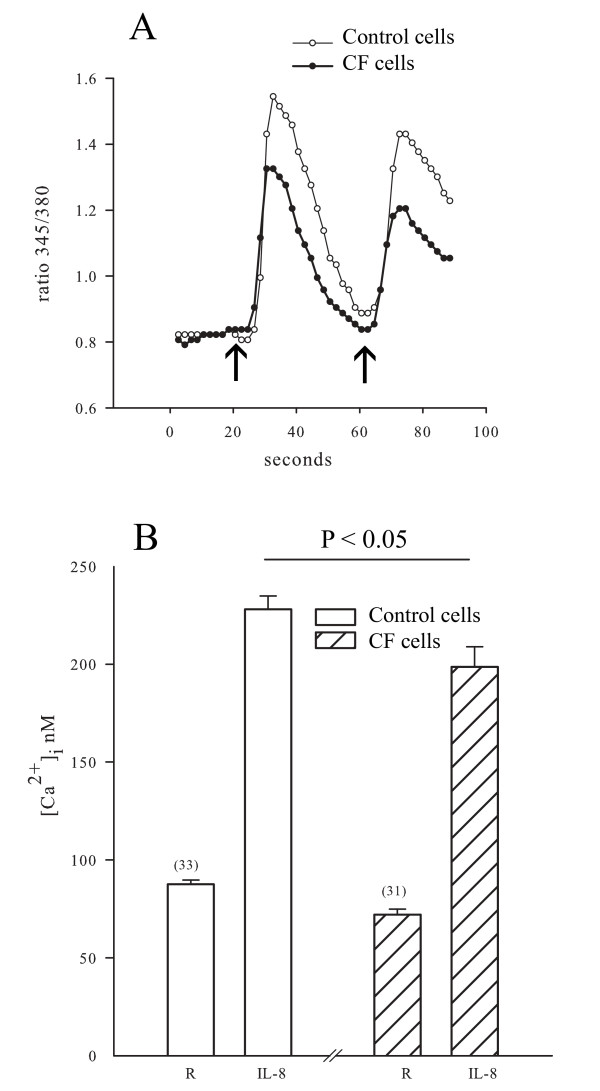
**Effects of IL-8 on [Ca^2+^]_i _in CF and control cells**. Cultured ASM cells from CF and control subjects were stimulated with IL-8 (100 nM). Illustrative examples of responses of a control cell and a cell from a CF-affected subject are shown in panel 1. The left hand arrow indicates the addition of IL-8 to the medium and the right hand arrow represents the addition of histamine (1 μM) to serve as a positive control. The resting [Ca^2+^]_i _(R) and the peak [Ca^2+^]_i _induced by IL-8 (IL-8) from the control (open bars) and the CF group (hatched bars) are shown. (n = 48 cells recorded on 6 different slides from 4 individuals in each group).

IL-8-induced Ca^2+ ^transients were measured in cells from control and CF-affected individuals. Figure 3a shows that IL-8 (100 nM) induced a rapid increase in the [Ca^2+^]_i_, which subsequently returned towards resting values. IL-8 increased the [Ca^2+^]_i _to 228 ± 7 nM in control cells, significantly greater than the value of 198 ± 10 nM in CF cells (p < 0.05; Figure 3b). The resting [Ca^2+^]_i _was 87 ± 2 nM in control cells and lower in CF cells (72 ± 2 nM; p < 0.05).

### IL-8 induced phosphorylation of myosin light chain_20 _(MLC_20_)

**Figure 4:**
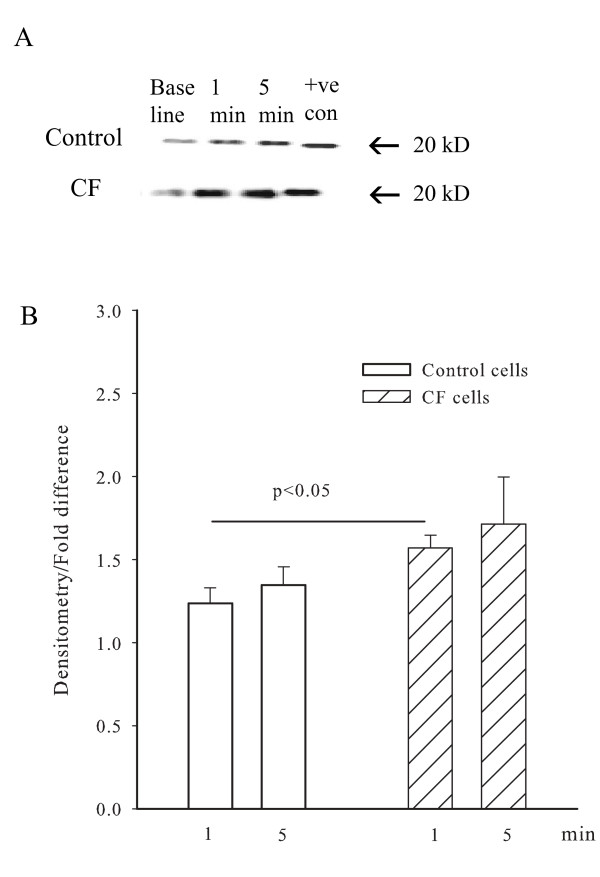
**IL-8 induced phosphorylation of MLC_20 _from CF and control cells**. Panel A shows representative blots of myosin light chain (MLC_20_) phosphorylation from CF and control cells. Bands correspond to baseline and IL-8 stimulation at 1 and 5 minutes. Thiophosphorylated myosin from chicken gizzard was used as a positive control (+ve con). Panel B shows the average increase in MLC_20 _phosphorylation (expressed as fold difference from baseline) in CF and control cells. The MLC_20 _phosphorylation from CF cells was significantly different from control cells at 1 minute after treatment with IL-8.

Western analysis was used to study the effects of IL-8 on the phosphorylation of MLC_20 _in CF and control cells. Figure 4 shows the extent of MLC_20 _phosphorylation in CF and control cells (panel A) under control conditions and after stimulation by IL-8 for 1 and 5 minutes. In panel B, the densitometry results (mean ± SEM) are expressed as the fold difference compared to baseline, the phosphorylation of MLC_20 _was increased at 1 minute after treatment with IL-8 consistent with activation of contractile signaling pathways and was significantly greater in CF cells (1.5 fold) than in control cells (1.2 fold). At 5 minutes, there was a further slight increase in phosphorylation, but the differences were not quite statistically significant between CF and control cells.

### Expression of myosin light chain_20_

**Figure 5:**
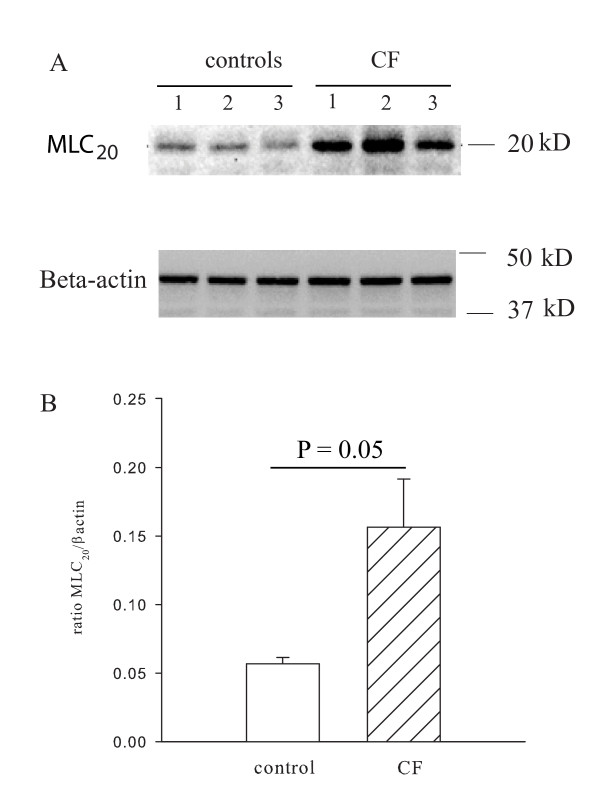
**Expression of MLC_20 _in CF and control cells**. Panel A is a representative blot for the expression of MLC_20 _in CF and control cells. Panel B. Mean densitometric values of MLC_20 _expression (corrected to β-actin) in CF cells is higher than control cells (n = 4 experiments).

Proteins were extracted from unstimulated CF and control cells and the expression of total MLC_20 _was determined by immunoblotting. Figure 5A shows the Western blot analysis for the expression of MLC_20 _protein in CF and control cells. Quantitative assessment with densitometry shows that the content of MLC_20 _was higher (Figure 5B, p < 0.05) in CF (15.7 arbitrary units) than in control cells (5.7 arbitrary units).

### Effects of IL-8 on migration of cells

**Figure 6:**
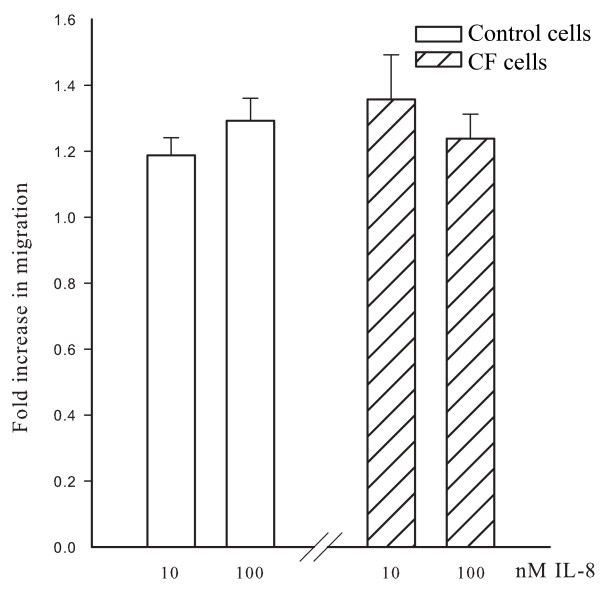
**The effects of IL-8 on ASM cell migration in CF and control cells**. Histogram illustrates the IL-8 induced migration of CF and control cells at concentrations of 10 and 100 nM. The data are represented as the fold difference compared to vehicle treated cells. There was no significant difference between CF and control cells.

A chemotaxis assay to IL-8 was performed and the results are shown in Figure 6 for the migration of CF and control cells in response to two concentrations of IL-8 (10 and 100 nM). The results are expressed as fold difference compared to vehicle treated cells. IL-8 stimulated the migration of both control and CF cells at concentrations of 10 nM and 100 nM compared to vehicle-treated cells. However, there was no difference in the migration rates of the two groups of cells.

### Effects of Il-8 on cellular proliferation

**Figure 7:**
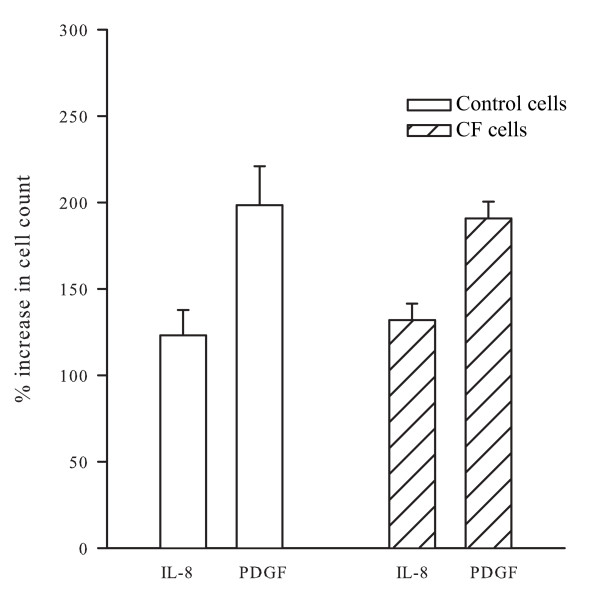
**IL-8 induces cell proliferation in both CF and control cells**. This figure shows the increase in cell counts measured with a haemocytometer (expressed as % of baseline) in response to IL-8 (100 nM) and PDGF (10 ng/ml) treatments. There was no significant difference between the CF and control cells.

Exposure to IL-8 evoked a modest proliferation of CF and control cells that was comparable in both groups: 132.0 ± 9.5% for CF cells (n = 4 independent experiments) and 123.2 ± 14.5% (n = 5 independent experiments) for control cells. PDGF was used as a positive control. It induced a robust proliferation (figure 7); the increase in cell proliferation following stimulation with PDGF was 190.8 ± 9.8% for CF and 198.6 ± 22.4% for control cells.

## Discussion

The results of this study demonstrate that IL-8 induces a greater contraction of ASM cells from CF patients compared to those of control individuals. The augmentation of ASM contraction is associated with a greater degree of phosphorylation of MLC_20 _with IL-8 and higher expression of MLC_20 _in CF cells. There was no difference in the expression of CXCRs between CF and control cells. Peak Ca^2+ ^release induced by IL-8 was decreased in CF ASM cells compared to control cells, an observation that was largely explained by a lower resting [Ca^2+^]_i_. A similar difference in Ca^2+ ^regulation in response to histamine has been observed in tracheal gland cells and in nasal epithelial cells of CF patients but the reason for this abnormality was reported as unknown [29,30]. Despite these alterations, neither migration nor proliferation was significantly different between the two groups. These results indicate that CF cells are hypercontractile to IL-8, an effect that is not observed in the proliferative and migratory responses.

Chronic infection and inflammation leads to loss of more than one third of the epithelium from both central and peripheral airways of CF patients [9]. As a result, the ASM cells are exposed to various inflammatory mediators such as TNF-α, IL-1β and IL-8. Cytokines such as TNF-α, IL-1β, IL-5 and IL-13 may modulate the contraction of ASM by indirect mechanisms through effects on cellular phenotype [31-33]. However chemokines such as IL-8 derived from inflammatory cells such as neutrophils [34], and perhaps from residual epithelial cells, may have direct effects on ASM as bronchonconstrictors because they act through G-protein coupled receptors linked to phospholipase C. Indeed IL-8 is a significant contractile agonist for human ASM cells [25]. In the current study we focused on IL-8 because of its importance for airway neutrophilic inflammation, which is a prominent feature of CF and is present also in some asthmatic subjects. The finding of the hypercontractile response to IL-8 may therefore have significance for the regulation of airway tone in CF affected subjects.

We tested the possibility that altered signaling mechanisms could account for the enhancement of the contraction in response to IL-8 by measuring the expression of CXCRs and the effects of IL-8 on [Ca^2+^]_i_. Flow cytometry confirmed our previous report of CXCR 1 and 2 expression in control cells [25], albeit at a lower level than in neutrophils. Our current results demonstrated comparable levels of expression of CXCR1 and CXCR2 between CF and control cells. This finding is not unexpected, given that the increase in responsiveness of CF cells to IL-8 was confined to its effect on the contraction whereas there were no differences in responsiveness as measured by migration and proliferation. We explored next the possibility that the enhanced ASM contraction in CF might be related to exaggerated increases in [Ca^2+^]_i_. Rather than the expected enhanced Ca^2+ ^transients in CF cells, fluorescence imaging of intracellular Ca^2+ ^showed that IL-8 evoked lower Ca^2+ ^transients compared to control cells. Next, we explored other mechanisms for the increased contraction of CF ASM cells, namely MLC_20 _phosphorylation. Our data showed that there was a greater increase in MLC_20 _phosphorylation in the CF cells compared to controls. However the increase in MLC_20 _phosphorylation was modest and less than the magnitude of the increased expression of MLC_20 _measured in the CF cells. In addition to its role in contraction, IL-8 can also trigger ASM to respond by proliferation or migration [25]. However the increased response of CF cells to IL-8 was not reproduced in relationship to other cellular functions such as chemotaxis and proliferation. The mechanistic link between the CFTR channel and the contractile properties of airway smooth muscle has not been established. However, Robert et al have reported that CFTR channels are present in rat vascular smooth muscle cells and that stimulation of the channels by specific CFTR agonists produces relaxation of pre-contracted vascular tissue [36]. Data from our laboratory show that CFTR channels are present and have functional effects on calcium signaling in ASM cells [37].

In conclusion, our findings show that the ASM cells of cystic fibrosis patients are more contractile than those of control subjects to stimulation by IL-8. This enhanced contractility appears to be attributable to phenotypic differences and could be responsible, at least in part, for the airway hyperresponsiveness and asthmatic diathesis observed in many of these patients.

## Competing interests

The authors declare that they have no competing interests.

## Authors' contributions

VG participated in the design of the study, carried out many of the experiments and wrote the manuscript. MCM established the cell culture and supervised the cell proliferation experiments. PF contributed to the cell culture. HVC carried out the western blot analysis, KS did the proliferation experiments. JA measured cell contraction. JGM conceived of the study, participated in its design and coordination, and helped to write the manuscript. All authors read and approved the final manuscript.
